# Prevention of MS Requires Intervention on the Causes of the Disease: Reconciling Genes, Epigenetics, and Epstein Barr Virus

**DOI:** 10.3389/fneur.2022.817677

**Published:** 2022-02-22

**Authors:** Patrick K. A. Kearns

**Affiliations:** ^1^Chromatin Lab, MRC Human Genetics Unit, Institute of Genetics and Cancer, University of Edinburgh, Edinburgh, United Kingdom; ^2^Anne Rowling Regenerative Neurology Clinic, Centre for Clinical Brain Sciences, University of Edinburgh, Edinburgh, United Kingdom; ^3^Department of Clinical Neurosciences (Neurology), Royal Infirmary of Edinburgh, NHS Lothian, Edinburgh, United Kingdom

**Keywords:** multiple sclerosis, genetics, environment, Epstein Barr Virus, virus, prevention, autoimmunity, neuroinflammation

## Abstract

Prevention of multiple sclerosis requires intervention on modifiable causes of the condition making it necessary to establish what those causes are. MS is often stated to be a polygenic disease, with causal contributions from environmental factors and gene-environment interactions, implying an additive and independent relationship of these factors. Mechanistically there are no independent contributions of genes or environmental factors to traits. This model is unrealistic but still useful and underlies the concept of heritability, a foundational parameter in population genetics. However, it perpetuates a debate on an irreconcilable dichotomy about whether MS is primarily genetic or environmental. In particular, epidemiological evidence now exists for a causal, possibly even necessary, role for Epstein Barr Virus in MS. The additive model makes it unintuitive to reconcile MS as a genetic disease but also independently a viral illness. In this perspective it is argued that starting from a realistic interaction only model, based on broadly accepted biological premises, and working forward to explain why the classical additive model gives useful results, there is actually no paradox. An integrated approach using population genetic studies, immunology and molecular virology offers a particularly promising route to establish the elusive role of EBV in MS pathology, as EBV is a large and complex virus and its latency, dysregulated in most EBV-related pathologies, is hard to study *in vivo*. This approach may offer a route to prevention of MS altogether.

## 1. Introduction

Prevention of multiple sclerosis (MS) occurring altogether, rather than prevention of MS disability by early diagnosis and effective treatment, would require intervening on the modifiable causes of MS, making it critical to establish what those are. However, fortunately much is now known about specific factors contributing to variation in MS risk (1).

Evidence for MS susceptibility being genetic is incontrovertible, and converges from many sources: aggregation of risk in families (2–5), robust genotype-phenotype associations for particular HLA alleles and over 200 non-HLA loci (6), from adoption studies (7), etc. Likewise, changing disease incidence over a small number of generations (8, 9), marked geographical variation in disease risk between and within countries coupled with findings of migration studies (10, 11), and serological [Epstein Barr Virus (EBV)] and robust lifestyle (smoking, adolescent obesity and vitamin D deficiency) associations indicate the importance of environmental factors even decades prior to diagnosis (12). Particularly, in the case of EBV infection, which is the only consistent, strong, and temporal association (genetic or environmental) that has thus far been suggested to be necessary for MS (13–25), the evidence for a causal relationship in at least most cases of MS is unusually strong as specific viruses are rarely *necessary* for clinical syndromes (and are perhaps never sufficient), so the apparent necessity of EBV in MS is strikingly unusual (26). The epidemiological association between EBV and MS has been reviewed previously (27–29), but it may be noted that genes and EBV do not appear to explain all of the epidemiological observations, and whilst the second half of this perspective will focus on opportunities to use genetic and immunological studies to understand the role of EBV in MS, this suggests that other important factors contribute to causing MS (12). Other “hits” may even be necessary.

Clearly both genes and environment are important, but currently it is usually not possible to intervene to modify an individual's genes to prevent disease and antenatal genetic screening as a method of prevention brings a myriad of serious ethical concerns. However, a powerful feature of understanding the genetic architecture of a disease is that it can lead to the identification of environmental factors which may be modifiable or can reveal the biological pathways that these factors are acting on. Drugs can be targeted to particular biology, and protective or harmful environmental factors can be epidemiologically identified by their genetic interactions and exposures modified. For a complex environmental factor like EBV infection, the pathobiology can still be an enigma even after the evidence for causation is strong. In this case, combining insights from genetics, virology, and immunology and reconciling the genetic and environmental factors into an integrated etiological model may be a very fruitful path to prevention of disease altogether.

## 2. The Root of the Nature vs. Nurture False Dichotomy

**Figure 1:**
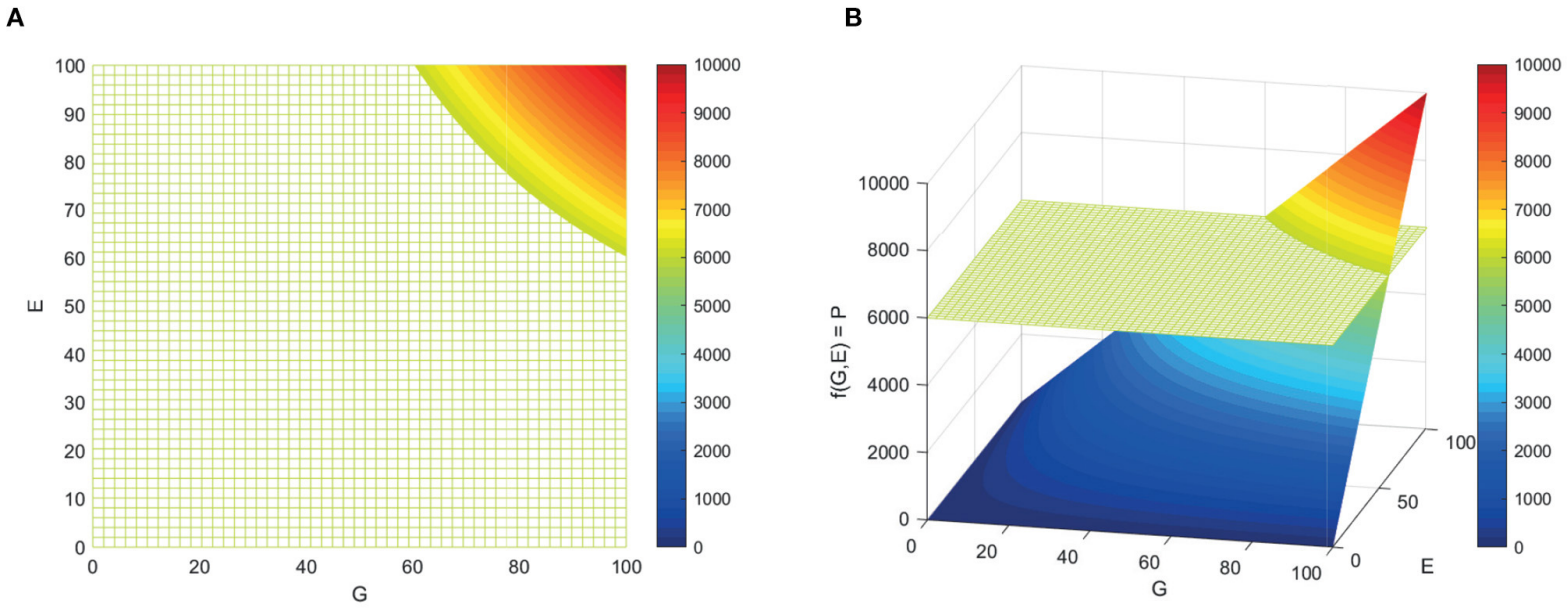
Surface representing the f(G,E) where phenotype is an interaction of genotype and environment. **(A)** From above. **(B)** Angled.

Acknowledging the epidemiological data, MS is often described as a complex genetic disease, with important causal contributions also coming from environmental factors and gene-environment interactions (30–37). This statement and paraphrases of it sound etiological but actually describe the classic additive model of population genetics which is primarily concerned with a related but different concept: partitioning the variation of a trait observed in a given population into the sources of that variation (Equation 1) (38, 39). Where total *variation* in phenotype (*P*) in the population, is statistically “explained” by the linear (independent or additive) combination of variation in phenotype due to genetic (*G*), and environmental factors (*E*), and their interactions (*G* × *E*). In the case of MS, the phenotype is risk or liability to develop MS [a continuous unmeasured (or latent) variable], where exceeding some threshold liability leads to disease penetrance (Figure 1). This partitioning also underlies the concept of heritability, which, for the purposes of statistical genetics, is defined as the proportion of phenotype attributable to the genotype term (*G*/*P*). Heritability, is a key parameter in population genetics, but “heritable” had a common language meaning dating to the fourteenth century in English and an established legal meaning relating to the inheritance of property before it had a technical one limited to partitioning statistical variation. Consequently, it is often confusingly used with imprecise or interchanging meanings (40).


(1) $$\begin{array}{l} P=G+E+\left(G\times E\right) \end{array}$$

To illustrate the problem with confusing this with an etiological model, we can consider the issue of gun crime. Gun crime is entirely an interaction between guns and criminals and there are no additional independent mechanistic contributions to gun crime from guns or criminals. All gun crime is 100% due to the interaction. However, if guns and criminals were modeled as independent factors to explain variation in gun crime, it might be possible to explain some % of the variation in gun crime across cities based on gun availability, even though it makes no mechanistic sense to say that (e.g.,) 60% of gun crime can be explained independently by the availability of guns and so gun crime is 60% caused by guns and 40% by criminals.

The fundamental problem with considering the model in Equation (1) as an etiological model, therefore, is that it implies that genes and environment contribute independently [in the first two terms (G) and (E) of Equation 1] when it is widely accepted that this is unrealistic. The addition of a more mechanistically plausible gene-environment (GxE) interaction term does not resolve the issue of the first two terms being mechanistically implausible. Population genetic studies typically partition both variation in phenotype due to genes and environment further: for example, into contributions from individual and shared environment; and into additive, epistatic, and dominant genetic influences. However, the initial partitioning into independent genetic and environmental terms is the root of the nature vs. nurture false dichotomy (41). This gives rise to an apparent paradox when a disease, such as MS, appears to have a strong genetic basis but also to be the result of a necessary environmental factor.

“*Without environmental inputs, your genome would have created nothing more than a damp spot on the carpet.”*

***Lykken, 1995***

“*if there is no environment, no organism can develop to display any phenotype whatsoever. Likewise, without a genetic constitution, there will be no organism.”*

***McLearn, 1964***

“*It is needless to insist that neither [nature nor nurture] is self-sufficient.”*

***Galton, 1874***

The above quotations and compilation of views from many authors on the merits of heritability and the nature vs. nurture debate is available in Sesardic, Making sense of Heritability, 2005 (42), illustrating more than a century of consensus on the unrealistic nature of this model.

In the next section of this perspective, a simple derivation of the additive model (Equation 1) is derived from a more biologically realistic model and the historical context and implications for understanding MS epidemiology are discussed. Thereafter, it is argued that by focussing the tools of population genetics, immunology, and molecular epidemiology on the difficult problem of latent EBV, an elusive epigenetic master manipulator of memory B cells, and by investigating MS as both a complex genetic disease *and* probably a viral pathology simultaneously, exciting opportunities for attempting MS prevention may arise.

## 3. Reconciling the Additive Model of Population Genetics With Molecular Genetics

### 3.1. Historical Perspective—The Additive Model Is Useful but Incomplete

The field of population genetics, and by extension MS genetics, is arguably founded on this model (Equation 1) and the heritability parameter, as they implicitly underpin the early work of pioneers Ronald Fisher and Sewall Wright. Particularly, Fisher's famous 1918 paper and subsequent work that set a mathematical foundation for reconciling Mendelian discrete units of inherited traits with the observations of continuous variation in traits like height measured by the early quantitative biometricians (43). Fisher's solution was to develop the statistical tools to partition the variance of traits in a population—variance could be quantified—into contributions from heritable (genetic) factors measured by the correlation between relatives and everything else. The concept and mathematics of correlation had been developed essentially for this purpose by Galton and Pearson, respectively. Fisher built on this, including by inventing the statistical technique of Analysis of Variance (ANOVA) which is based on the linear or additive segregation of contributions to variance. Wright's application of his method of path analysis, the ancestor of many modern techniques of causal inference, is analogous to Fisher's ANOVA in this respect as both chose to partition genes and environment as though they were independent (44) and all work based on the concept of heritability has carried the underlying partitioning since.

Initially, this approach was probably adopted because very little was known about the biochemical nature of genes in the era prior to the discoveries of Avery et al. (45) and Crick and Watson (46), and so it was perhaps as reasonable a choice as any other model. Although the prejudices of the scientific establishment were almost surely also relevant and fueled the eugenics movement of the time. If Fisher and contemporaries thought about the biological nature of the units of heredity whilst establishing the mathematics in the first four decades of the twentieth century, they probably thought they were likely to be proteins, discrete material units, capable of extraordinary complexity, that might actually have exerted independent effects to external environmental factors. However, biologists criticized this early mathematical work for being overly theoretical, and subsequently, when years later the nature of genes as code for biochemically interpreting the environment was discovered, as mechanistically unrealistic. However, by that time, decades of theory based on the additive model had proved to work successfully enough that attempts to point out it was unrealistic did not dissuade its use.

“*I do not feel that this kind of work affects us biologists much at present. It is too much of the order of problem that deals with weightless elephants upon frictionless surfaces, where at the same time we are largely ignorant of the other properties of the said elephants and surfaces.”*

***Biologist R. Punnett's lukewarm review of Fisher's now famous 1918 paper at the Royal Society of London*.**

### 3.2. All Models Are Wrong, but If This One Is Unrealistic Why Is It Useful


(2) $$\begin{array}{l} P=G\times E \end{array}$$

To address the realism problem, that biology supports interaction only (Equation 2) and not independent contributions (Equation 1), population genetics texts offer the disclaimer that additive model and heritability tells us not about individuals or mechanistic causes but rather about the causes of variance in the specific populations *n* > 1 under investigation and that these findings are not necessarily valid if generalized to other populations or to individuals (as can be understood in the gun crime analogy) (40, 47).

But the idea that the sign can change from interaction (multiplicative) to addition just because the population *n* > 1 is surprising. The simplest population is two individuals, and Equation (1) is unrealistic here too, as the phenotypes of the population are the sum of the two individual phenotypes, generally $P=\overset{n}{\underset{i=1}{\sum }}\left(G_{i}\times E_{i}\right)$. So when is a population big enough for the additive approach to work and why? Perhaps more importantly, this risks underselling the mechanistic insights that are gained from studying the heritability of traits in populations. Genes that are associated with variance in the risk of MS in large GWAS, do inform as to the biological pathways mechanistically important for MS pathology in individuals because genes and environment act on individuals and do not have effects on the phenotypes in a population except via the sum of their effects on individuals. If a variant in a gene causes variation at the population level, it can only do so by being a mechanistic cause of the trait in at least some individuals.

Causes of variation in a population are, therefore, a subset of the mechanistic causes of that trait. However, other important mechanistic causes may not be responsible for any variation, for example, because they are ubiquitously experienced, or strongly associated with other causal factors that negate their associations: just as association does not imply causation, causation does not imply association.

The analogy of gun crime is useful because it highlights where the model stops being useful. If we did not know the mechanistic nature of gun crime, we might infer the importance of guns from discovering that their availability explained some of the variation across cities, whereas variation in access to spoons, kitchen chairs, or other household objects does not. But, if everyone had abundant and equal access to guns we would need a different approach despite guns still being a necessary cause of gun crime. Similar inferences can be drawn from the study of genetic variation and association with traits of interest at the population level for diseases like MS where the causes are less obvious. However, caution is particularly necessary not to discount the contribution of genes that are tightly conserved and environmental exposures that are ubiquitous or nearly so.

### 3.3. Deriving the Useful Additive Model From the Realistic Interaction Model

**Figure 2:**
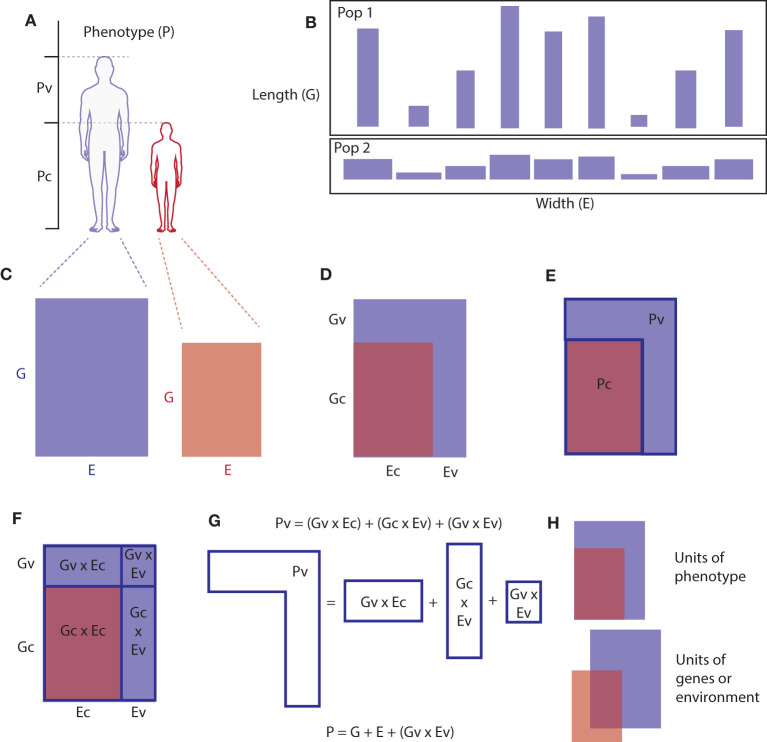
Geometric representation of the partitioning of causes of a phenotype into genetic and environmental causes that are causing variation in the population and non-varying causes. Just as genes and environment are inseparable in causing phenotypes, the area of a rectangle cannot be considered to be mainly the product of either its length or width. However, if an individual's genes are represented on one axis (length) and their environment on an orthogonal axis (width) and phenotype represented by the area, then in a population of such individuals, most of the variation in area(phenotype) across the population can come from variation on one of the axes [e.g., the length (genes) axis]. For the simplest possible population, two individuals (two rectangles), we can see visually how Equations (1) and (3) are related. **(A)** Partitioning phenotype in a population (two individuals), phenotype of interest is represented as height, but any phenotype could be partitioned as such, **(B)** two populations of rectangles where the variation in area across the population is mostly the result of variation in length rather than width. **(C)** Phenotype represented as the product (area) of two necessary interacting factors. **(D–F)** Partitioning causal genes and environmental factors into common and varying contributions. **(G)** Decomposing the genetic and environmental contributions to phenotypic variation (Pv) into the additive model. **(H)** This model only works when the G and E terms are measured in terms of phenotype, when measured in units of genes or environment themselves the model would require a different (multivariate) model.

Starting from the premise that MS is exclusively the product of gene-environment interactions, gives the interaction model in Equation (2) which captures the reality of mechanism but otherwise is not useful. Working forward to derive the useful additive one (Equation 1) gives an insight into why the additive model gives useful results and makes the meaning of parameters derived from it (like heritability) more intuitive. A geometric representation of this is presented in Figure 2 for the simplest possible population (*n* = 2). First, if we consider that all the causal factors for any phenotype can be partitioned into those that are also causing variation in the population and those that are causal but not causing variation (for example, because they are ubiquitous), then we can model the total (varying and non-varying) effects (P) and causes (G,E):


(3) $$\begin{array}{l} P_{t}=G_{t}\times E_{t} \end{array}$$

where the subscript (t) denotes the total effects (P) or causes (G,E) for each term. Each term can then be partitioned into mutually exclusive and jointly exhaustive sets based on whether they are also observed to be varying (P) or causing variation (G,E) in the given population (v, varying) or not (c, constant). So the total phenotype *P*_*t*_ is the sum of the phenotype that is varying *P*_*v*_ and that which is not varying (common to everyone in the population) *P*_*c*_, and similarly *G*_*t*_ is the sum of causal genes that are also causing variation *G*_*v*_ and those that are not causing variation *G*_*c*_, and so on for environment. As below (Equation 4),


(4) $$\begin{array}{l} P_{t}=P_{c}+P_{v};G_{t}=G_{c}+G_{v};E_{t}=E_{c}+E_{v} \end{array}$$

If we then say that we are only (for practical reasons) interested in the part of the phenotype that is varying, because that can be quantified using the units of population variance, then we need to subtract *P*_*c*_, which will be only the component with no contributions to variation (v terms) (Equation 5).


(5) $$\begin{array}{l} P_{c}=G_{c}\times E_{c} \end{array}$$

and so by rearranging the first Equation in (4), and substituting the right hand side of Equation (5), we can get,


(6) $$\begin{array}{l} P_{v}=P_{t}-\left(G_{c}\times E_{c}\right) \end{array}$$

and by replacing *P*_*t*_ with the right hand side of (Equation 3) we get,


(7) $$\begin{array}{l} P_{v}=\left(G_{t}\times E_{t}\right)-\left(G_{c}\times E_{c}\right) \end{array}$$

which expands using definitions for *G*_*t*_ and *E*_*t*_ in (Equation 4) to,


(8) $$\begin{array}{l} P_{v}=\left(G_{v}+G_{c}\right)\times \left(E_{v}+E_{c}\right)-\left(G_{c}\times E_{c}\right) \end{array}$$

and after expansion of the first two bracketed terms, the constant terms (*Gc* × *Ec*) cancel out. Giving,


(9) $$\begin{array}{l} P_{v}=\left(G_{v}\times E_{c}\right)+\left(E_{v}\times G_{c}\right)+\left(G_{v}\times E_{v}\right) \end{array}$$

If we take any given population as having fixed conditions (for example, because they've already happened), then the constant terms can be treated as constants and ignored (as long as we remember that they do exist and may not necessarily be the same level or constant in other populations, see Figure 2B), then we get back to the classical additive model (Equation 1). All terms can then be measured or calculated in units of variation/deviation from the mean phenotype to allow them to be quantitatively interrogated. This is equivalent to mean centering (subtracting the mean value of phenotype (μ), scaling by standard deviation, and adding some random measurement error (ϵ), giving the familiar linear model:


(10) $$\begin{array}{l} P_{v}-\mu =\left(G_{v}-\mu \right)+\left(E_{v}-\mu \right)+\left(\left(G_{v}\times E_{v}\right)-\mu \right)+\epsilon \end{array}$$

This demonstrates that the additive model (Equations 1 and 10) is an incomplete simplification of a realistic interaction model (Equation 9). It will be generalizable to populations other than the one it is fitted on only in the special circumstance where the environmental causes and genetic causes are the same and are partitioned in the same way. That is, when the same genetic and environmental causes are present and are also contributing to variation or not in both cohorts. Whether this is the case or not depends on what these factors are and how similar exposure profiles are in the populations in question. This implies that heritability is actually best understood as the proportion of variation explained by the interaction of genes that are causing variation in the population with ubiquitous causal environmental factors, not as the proportion of variation explained by genetic variants acting independently.

### 3.4. Implications of This Model to MS Epidemiology

The first implication is that if a trait such as MS were to be 100% heritable it would only mean that the environmental factors causing it are not causing variation, not that it is a purely genetic disease and environmental causal factors are unimportant. Rather they will surely exist, but probably will have been ubiquitous or near ubiquitous, but without knowing what they are it is impossible to say whether they are modifiable, necessary for some other physiologically essential reason, or unavoidable environmental factors. Therefore, the necessity of a near ubiquitous virus like EBV is no barrier to observing a heritability considerably higher even than that which is actually observed in the case of MS. In fact, the more ubiquitous a necessary environmental factor is the higher the heritability will tend to be and the more aggregation in families that should be expected if causal genetic variants are also contributing to variation in the population. Therefore, there should be no theoretical conflict in accepting MS as a complex genetic disease and also the consequence of a viral infection simply on the basis of its heritability or aggregation in families.

The fact that the heritability of MS is much <100% probably speaks to the importance of other less ubiquitous environmental factors (beyond EBV) causing variation mostly by interacting with conserved genes *E*_*v*_ × *G*_*c*_ (the equivalent term to *E* in Equation 1) although it could in theory be possible that varying exposure to different strains of EBV (some pathogenic and some not) or timing of infection could account for some reduction of heritability. Whilst hypothetical, both have been suggested (48–50), and were it to be the case would add another layer of complexity, as when EBV is acquired in childhood, it is typically acquired from within the family unit. Therefore, as with human genes, EBV is also inherited identically by descent to an extent. This would mean that some aggregation due to shared ancestry could be mis-attributed to shared human genes, where the correlation between relatives in phenotype is also affected by the correlation between relatives in the pathogenicity of the strain of virus or timing of infection.

A second related implication, is that changes in exposure to environmental factors over time or space will affect estimates of heritability even where the frequency of gene variants are unchanged. Consider two otherwise identical hypothetical populations, where in one 30% of individuals smoke, and the other everyone does. Because smoking is an established risk factor for MS, the effect of increased smoking in the all-smoking cohort would be to inflate the (*Gv* × *Ec*) term (Equation 9). In the classic additive model (Equations 1 and 10) this term is thought of as representing the genetic influences *G*. Therefore, an increase in environmental exposure, would counter-intuitively be reflected in higher estimates of genetic influence and heritability in the all-smoking cohort, and lower estimates of the influence of environmental factors and gene-environment interaction. At least, that is, if taking Equations (1) and (10) at face value (forgetting that each term does in fact represent a mechanistic *G* and *E* interaction). Higher estimates of heritability in locations with higher MS incidence has been demonstrated, and the more realistic model (Equation 9) explains the counter-intuitive but probably correct conclusion that the higher heritability in higher incidence populations is likely to reflect a higher burden of environmental exposures (51, 52). Although strictly speaking the heritability will increase when the environmental exposure is more constant and this could be higher or lower mean exposure or the same so long as less variation occurs (consider populations where 25% of individuals smoke one packet of 20 cigarettes per day vs. populations where 100% smoke either 1, 5, or 40 cigarettes per day). The latter three populations would all be expected to have higher heritability than the first cohort all else being equal.

A further implication is that despite replication in large genetic studies being important in eliminating associations caused by biases arising from observational nature of the study design, genotype-phenotype associations that do not replicate across cohorts may include some of the most interesting real causal associations. Because where genotype-phenotype associations do not replicate due to differences in environmental exposures captured under *Ec* between cohorts, it suggests that these environmental exposures are probably modifiable (despite not causing variation in either cohort independently). This could occur if, for example, a causal environmental factor is ubiquitously present in one cohort but not another. This may be of particular interest in the case of MS where there is wide variation in incidence between nations/regions/cultures (where ubiquitous exposures could plausibly differ), and where environmental exposures have been suggested to vary “at the population level” (31). If the two hypothetical near-identical-except-for-smoking cohorts in the previous paragraph, had smoking prevalences of 0% (not 30%) and 100%, then smoking would differ at the population level (i.e., between the populations) and not contribute to variability within either cohort. However, some of the gene-phenotype associations present in the all-smoking cohort [captured in the (*G*_*v*_ × *E*_*C*_) term], but not replicating in the non-smoking cohort would reveal genes that mediate the causal effect of smoking.

Critically, all terms (P,G,E) on both sides of these equations are in units of variation in phenotype, which may be important for considering how specific causal factors are divided between the terms. This means that a given environmental or genetic factor may fall into more than one category (c) or (v) in differing proportions, because the model would have to be specified differently (from the bottom up) if each gene or environmental factor were to be partitioned into one or other term (Figure 2H). For example, in a matched case-control GWAS study for MS 90% of controls will have EBV, and 100% of the cases will (if EBV is necessary for MS), so 90% of matched pairs will be concordant for the apparently necessary factor, the virus. EBV would be reflected in the *E*_*C*_ terms of Equation (8) for 90% of pairs, and for the 10% will contribute to the *E*_*V*_ containing terms. This would also mean that 10% of controls in these studies are therefore not at risk of MS due to being EBV naive, meaning that all estimates for genetic variants associated with MS will have effect sizes diluted or biased toward a null effect because some of the controls are not at risk regardless of their genetic risk. Thus, if EBV is a necessary cause of MS, then the effect sizes genome-wide for SNP associations with MS are likely to be systematically underestimated by 10%. As the underlying models differ, this may explain some of the missing heritability phenomenon that occurs when top down and bottom up approaches to calculating heritability do not agree.

## 4. Epstein Barr Virus: A Master Epigenetic Manipulator of B Cells

### 4.1. Epidemiological Framework for Identifying Causal Associations

The epidemiological literature has been reviewed multiple times in the context of converging evidence, and is at least consistent with MS being a complication of EBV infection (27–29, 53, 54), but no definite counterfactual or experimental evidence exists to prove or disprove whether EBV causes MS, as no antiviral drug is known to clear latent EBV infection and as yet no vaccine protects from infection. However, classical causal theoretical frameworks can be considered strongly supportive. For example, the first four criteria set out by Bradford-Hill as the most important for judging an epidemiological association to be causal, overlap the criteria of a founder of causal philosophy, David Hume (55, 56). Hume identified strength, consistency, and temporality (cause before effect) as hallmarks of causal associations, and this insight underpins Bradford-Hill's attempt to establish a framework for epidemiological causal inference. Bradford-Hill made it clear that his nine criteria were not a checklist, but an ordered list with Hume's criteria three of the most important four determining when an association is likely to be due to cause and effect (56):

**1. Strength of association** (measured as ratio, not as an absolute difference): “*First upon my list I would put the strength of the association... in this situation I would reject the argument sometimes advanced that what matters is the absolute difference between the death rates of our various groups and not the ratio of one to the other. That depends upon what we want to know. If we want to know how many extra deaths from cancer of the lung will take place through smoking (i.e., presuming causation), then obviously we must use the absolute differences between the death rates... But it does not follow here... that this best measure of the effect upon mortality is also the best measure in relation to etiology. In this respect the ratios... are far more informative”*

The EBV-MS association is very strong, such that cases of EBV-naive MS, if they exist, are extremely rare, whereas 5–10% of the adult population will be EBV-naive (21). In fact, if one accepts that EBV *is* found in 100% of individuals with MS in large cohorts, if sufficiently sensitive methods are used (21), but only in 90% of healthy controls, then the point estimate on the odds ratio (odds of disease given exposed/odds of disease given unexposed) would be infinite and the lower bound on confidence very large. Given small biases cannot cause large effect sizes, Bradford-Hill argues that on this criteria alone, in the face of such a strong association, similar to that seen in the association between smoking and lung cancer, a non-causal explanation for the association, if it exists, should be obvious:

“*Though there is good evidence to support causation it is surely much easier in this case to think of some feature of life that may go hand-in-hand with smoking—features that might conceivably be the real underlying cause or, at the least, an important contributor, whether it be lack of exercise, nature of diet, or other factors. But to explain the pronounced excess of cancer of the lung in any other environmental terms requires some feature of life so intimately linked with cigarette smoking and with the amount of smoking that such a feature should be easily detectable. If we cannot detect it or reasonably infer a specific one, then in such circumstances I think we are reasonably entitled to reject the vague contention of the armchair critic “you can't prove it,” there may be such a feature.”*

**2. Consistency:** “*Next on my list of features to be specially considered I would place the consistency of the observed association”*

The EBV-MS association has been observed consistently across studies in various patient groups, geographies, ethnicities, ages, sexes, and sub-types of MS (14, 25, 57). This is important because the consistency across multiple studies improves the statistical (frequentist) confidence in the association, making it less likely to have occurred by chance, but also limits the alternative explanations to biases that would also be present across these multiple diverse settings.

**3. Specificity:** “*the specificity of the association, [is] the third characteristic which invariably we must consider”*

The lack of a consistent association with other saliva-transmitted viruses (e.g., CMV) reduces the probability that some large bias accounts for the observation and restricts the kind of bias that could be responsible. For example, this persuasively excludes many other “features of life” that could potentially result in higher exposure to EBV as an explanation because these features would also be non-specifically associated with exposures to other infectious agents and the necessary association observed is EBV specific (17, 25).

**4. Temporal relationship:** “*My fourth characteristic is the temporal relationship of the association”*

Evidence of EBV associations with MS in serosurveys has critically also been demonstrated to be temporal (EBV always before MS) in longitudinal studies (14, 18, 20, 58). This is further persuasive of a causal effect, and excludes many person-specific potential biases and reverse causality, e.g., shared susceptibility to EBV and MS, as it demonstrates that even for those who develop the disease (and so are susceptible to it) the risk of MS is extraordinarily low or nothing in these individuals prior to them contracting the virus.

Therefore, whatever the cause of MS is, it will ultimately have to explain why such a strong, consistent and temporal association with EBV is observed. If some bias accounts for the association making EBV simply a bystander, rather than a cause, then it begs the question, why is it not obvious what the explanation is?

### 4.2. Biological Plausibility for a Causal Association

The epidemiological case is further bolstered by the biological plausibility of EBV (plausibility being one of Bradford-Hill's lesser criteria) as both a known cause of serious pathology and specifically as a cause of autoimmunity. Transient autoimmunity is recognized to occur at the time of primary EBV infection with infectious mononucleosis when the virus replicates to extremely high levels before the host adaptive immune system recognizes its presence (59–63).

In addition, several aspects of the natural history of MS fit the biology of EBV. As a persistent herpes virus infection which periodically reactivates from a quiet latency in a fluid compartment, the dissemination in time and space of neuroinflammatory attacks occurring over decades in persons living with relapse-remitting MS fits strikingly well. In addition, the site of life-long viral latency is strictly memory B cells, which are now known to be important for pathogenesis and a therapeutic target (64–67). Thus, a reasonable index of suspicion based on biology of the virus (prior probability) combined with a suitable likelihood from epidemiological evidence makes EBV a credible cause of MS.

EBV is a remarkably-successful, large, double-stranded DNA gammaherpes virus that for most of human history has been practically ubiquitous, now being only nearly ubiquitous by adulthood in high-income nations (67). In recent generations, EBV has transitioned from millions of years of equilibrium to a non-equilibrium virus with a reproductive number less than one (*R* < 1), reflected in the rising average age of infection and new evolutionary pressures. In low-income settings, almost everyone is still infected in early childhood, but in high income settings some 10–50% of individuals escape infection in childhood with most of these individuals acquiring the infection later in life (sometimes experiencing infectious mononucleosis) such that 90–95% of people are infected eventually. The virus has co-evolved with its human hosts in this ecological niche for millions of years (67, 68).

**Figure 3:**
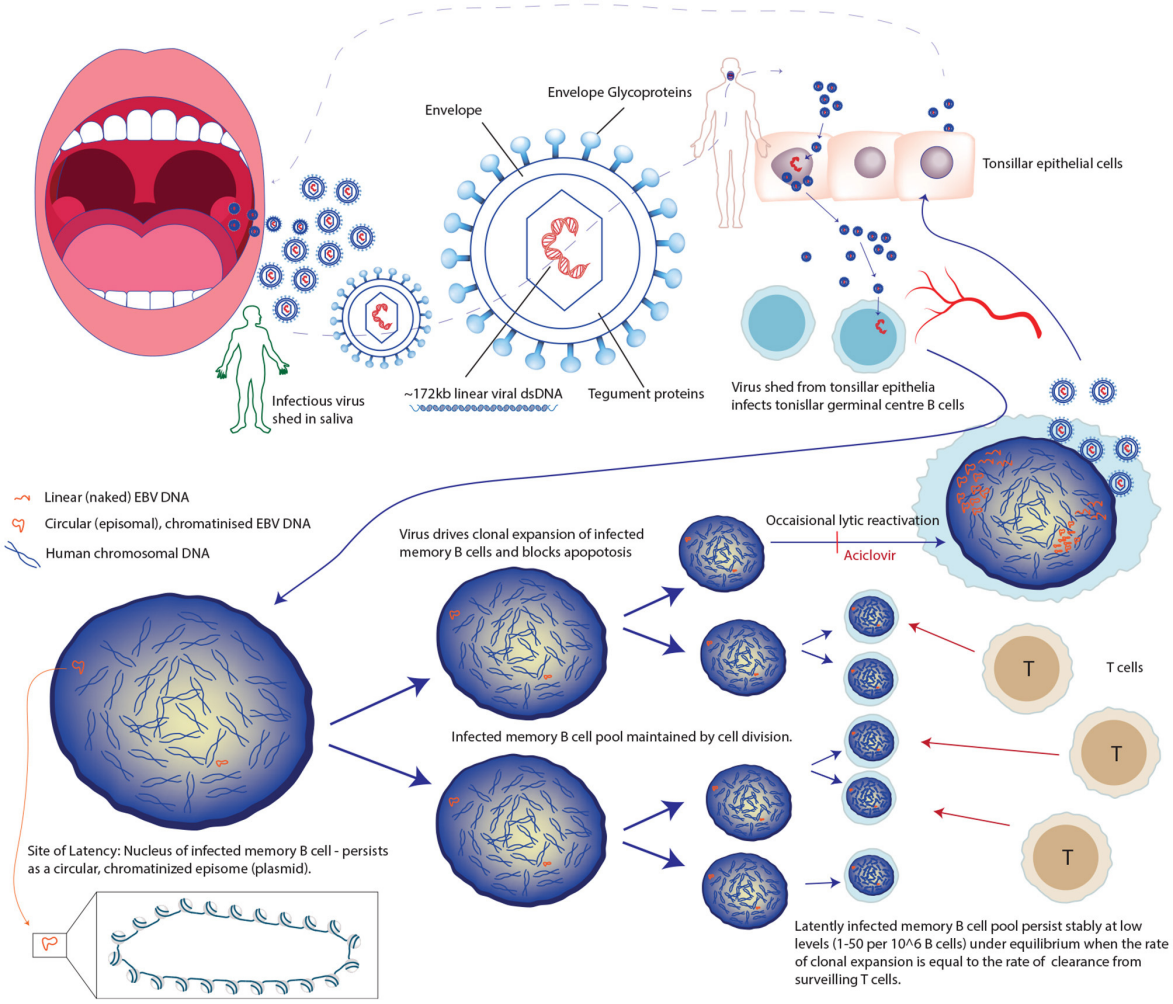
Unusual life cycle of Epstein Barr Virus explains its success and association with cancers and immune pathologies.

Consequently, EBV has specialized to make use of several unusual aspects of B cell physiology (Figure 3). Rather than rapidly replicating itself thousands of times (in the lytic cycle) on first infecting a B cell, as occurs with most viral infections, it expands the latently infected pool using a specialist repertoire of genes which drive the cell cycle, manipulating the pathways that B cells use to clonally expand and select for immunity in response to external stimuli (such as recognizing their cognate antigens) (67, 69). In order to do this EBV has acquired mimics of critical B cell specific signals, which essentially renders the infected B cells autonomous of T cell help and antigen (69, 70). Whilst manipulating the cell, the virus hides in the nucleus as a circular pseudochromosome (or episome), chromatinized and epigenetically marked, but not integrated into the human chromosomes. It is copied once and only once per cell cycle and faithfully segregated to daughter cells using only the host cell's replication machinery and a single viral protein. In effect, EBV immortalizes these cells whilst masquerading as a human chromosome and, transcribing only a very tightly controlled subset of its genes. This tight manipulation of both viral and cellular gene expression is a masterclass in epigenetic regulation, involving DNA methylation, histone modifications, and a complete 3-dimensional re-organization of chromatin environment within the nucleus of infected cells (71–73). This unusual strategy explains why so many EBV-related pathologies are lymphoproliferative, and why problems of dysregulated latency are common features of most EBV-related pathologies. Because as successful as this strategy is, driving cells to clonally expand and rendering them autonomous is risky, with a clear line of site to cancer and to autoimmunity. Thus, the absence of pharmacological tools that target latency, rather than lytic infection, is unfortunate (Figure 3).

Controlling latently-infected EBV is energy-intensive and a precarious immunological task, as evidenced by the 1~,000-fold increase in EBV-related malignancies in persons with CD4+ immunosuppression as a result of HIV infection, increase in latent cell number before and after some forms of (T cell affecting) immunosuppression, and the fact that a mutation in a single gene on the X-chromosome that leads to a defect in a protein important for T cell signaling, causes a lethal form of fulminant infectious mononucleosis called X-linked lymphoproliferative (XLP) syndrome in males after infection with EBV (67, 68, 74).

**Figure 4:**
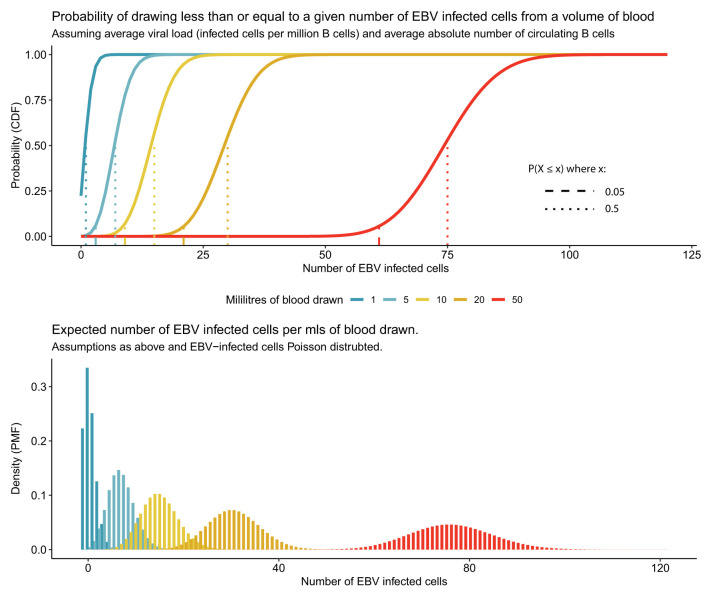
Rarity of EBV infected cells in peripheral blood makes studying latent EBV challenging. Average person has 500,000 latently EBV-infected cells in circulation, resident in a small subset of circulating B cells. For different quantities of peripheral blood the 95 and 50% (expected mean number) probability of EBV-infected cells are represented for an individual with the average viral load (5 per million B cells infected) and average absolute number of circulating B cells (3 × 10^5^ per ml).

**Figure 5:**
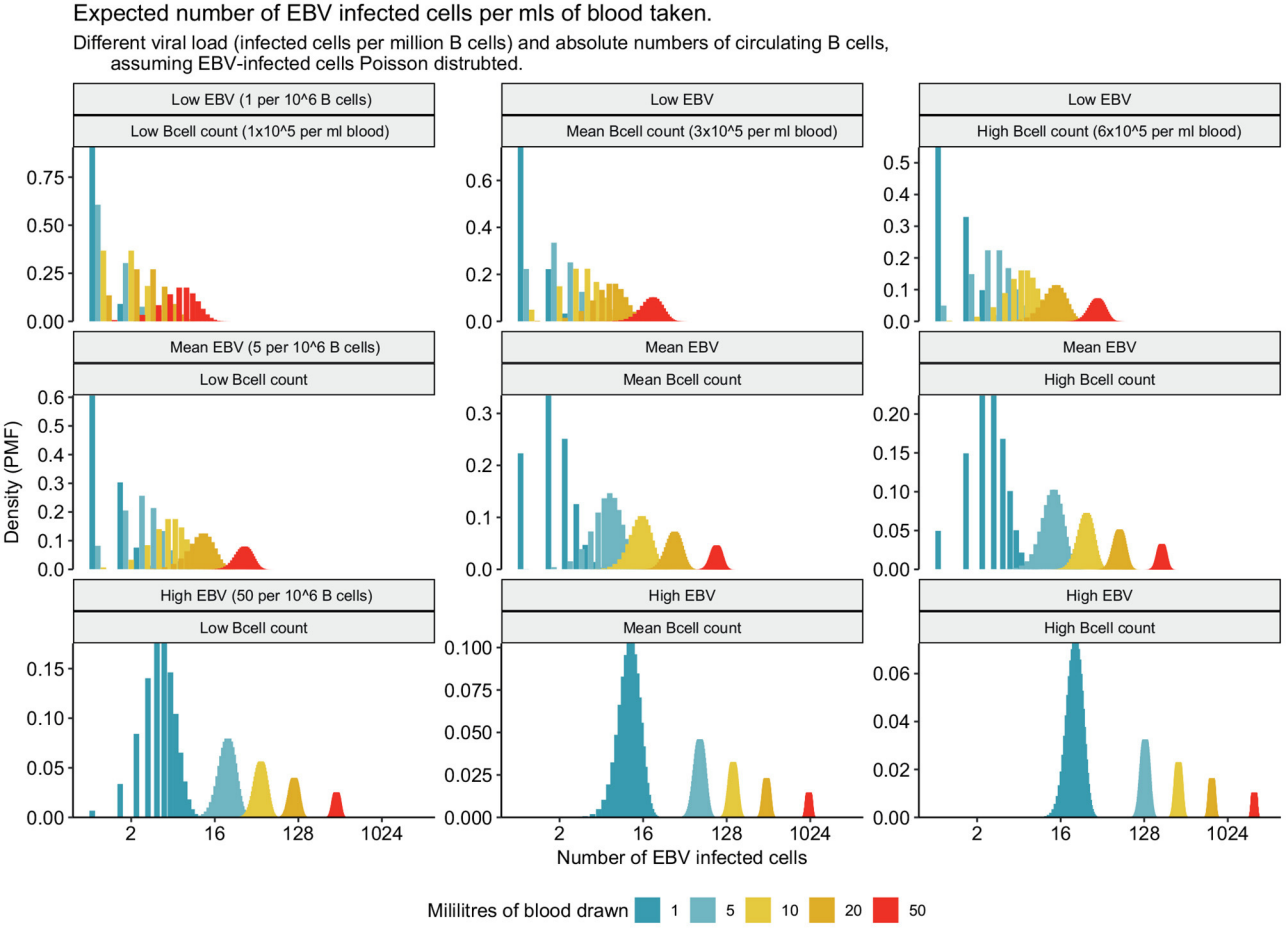
Frequency of EBV infected cells for low, mean, and high B cell counts and EBV viral loads.

Unfortunately, studying the epigenetics of latency in MS patients and healthy controls, outwith the context of lymphoproliferative conditions, is challenging due to the virus being in equilibrium with T cell surveillance which maintains a low number of infected B cells (Figure 3). During established latency, the infected cell pool is a very small subset (in the region of 1–50 infected cells per million) of the total circulating B cells meaning that a large volume of blood needs to be collected to be sure of collecting even one virally infected cell. For example, an individual with a low normal B cell count and a low proportion of virally infected cells could have as few as five infected memory B cells in 50 mls of peripheral blood (Figures 4, 5) (75). Further, when EBV amplifies itself by switching to lytic production, it linearizes and strips its DNA of its epigenetic marks for packaging into viral particles, essentially wiping it clean (68). Technologies are improving potentially allowing for single cell and rare cell approaches to address this, but it remains a technical hurdle.

For these reasons, using powerful but indirect methods (such as population genetics and immunology) to study the role of latent EBV in MS pathophysiology may yield valuable clues as to the nature of the pathology. Observations that many of the significant MS SNP-associations overlap with EBNA-2 transcription factor binding sites, for example, is interesting because this agrees with observations that anti-EBNA2 antibodies are part of the subset of EBV proteins that show a raised antibody profile in MS (23, 76–79). If these studies are interpreted as converging on a role for EBNA-2, then this gives an insight into the nature of the dysregulation of latency, as EBNA-2 is an essential for regulating viral latent gene expression and for EBV-driving lymphocytes into the cell cycle. Expression would be expected to increase EBV-infected cell number or turnover (67, 69).

A further example might be drawn from a study of cell-type specific transcriptomics (80), which identified genes involved in Neddylation as differentially expressed in the lymphocytes (T cells) of MS cases vs. controls. This is striking because Neddylation is evidently also a critical process for EBV and other herpes viruses, so much so that the EBV carries its own deneddylase enzyme amongst its tegument proteins, and antibodies against this viral protein (encoded by gene BPLF1) have recently been identified as a predictor of EBV viral load using an unbiased antibody screening method (80–82). Pharmacological targeting of viral enzymes is a potential therapeutic strategy, and so may be another fruitful avenue for further exploration. Intriguingly, the same drug targeting this pathway, has been proposed for trials in MS and in treating a human herpesvirus (81).

### 4.3. EBV-Focussed MS Prevention Possibilities

Even before the pathophysiological role of EBV in MS is established, it is possible to speculate generally on what a successful EBV-directed preventative strategy might look like.

#### 4.3.1. Vaccination

Vaccination aimed at preventing infection with EBV would be clinically useful in a number of contexts beyond autoimmunity, for example, in preventing infectious mononucleosis or post-transplantation. These other indications may substantially de-risk the investment required to develop such a vaccine, given the EBV-MS association is not universally accepted as causal. In the context of prevention of MS, targeted vaccination for EBV-negative young adults who are at risk geographically and/or genetically (assessed either by polygenic risk or as a result of family history) of MS may be particularly worthwhile as using sophisticated models of known predictors of MS risk it has been demonstrated that individuals can be identified with much higher than population risk of MS (83). Targeting young adults pre-college, as is practized for meningococcal vaccination, may be particularly worthwhile given the high hazard rate of EBV exposure at this life stage.

Initial vaccination attempts for EBV used a recombinant peptide vaccine aimed at a single immunodominant glycoprtein (gp350) (84). Unfortunately, this was unable to prevent infection, however, a Phase I trial of a combination mRNA vaccine for six surface glycoproteins has recently entered clinical trials (clinicaltrials.gov/ct2/show/NCT05164094). As a large DNA virus, EBV mutates extremely slowly and has <1 transmission opportunity for selection per human lifetime, thus this progress is extremely exciting and whilst primary outcome of the trial is the prevention of infectious mononucleosis and EBV infection, if the vaccine is effective at preventing the latter then this immediately would open the door to prevention of MS in EBV-naive individuals.

Vaccination may also be fruitful in those already infected with EBV. One school of thought is that to maintain a latently infected pool of lymphocytes EBV has to maintain a low level of continuous new infection. In this circumstance vaccines aimed at reducing new infection could be of benefit. However, an alternative strategy would see vaccination aimed at restory cellular immunity and improving anti-viral T cell surveillance and latent-gene expressing cell clearance. This is roughly the principle on which the shingles vaccine targets another member of the human herpes family, to control latent infection. Thus, vaccination may not only be useful in naive individuals but could perhaps be a useful strategy to help rebalance the virus-immunity equilibrium in persistently-infected individuals prior to or even after the onset of MS.

#### 4.3.2. Anti-virals and Anti-cancer Drugs

Latent EBV infection in growth transformed (rather than resting) cells profoundly alters the nuclear organization, cellular gene expression and the metabolism of infected cells in a manner similar to cancer. EBV-infected dividing cells, for example, show aerobic glycolysis a hallmark of cancer cells (85, 86). Where EBV-infected cells behave or can be triggered to behave differently from normal, healthy, uninfected cells, opportunities may arise for drugging pathways that infected cells are particularly sensitive to. In addition, many anti-viral drugs have broad activity beyond the classes of virus that they are licensed for use in. Therefore, there may be as-yet overlooked anti-EBV efficacy of other licensed or experimental drugs which may be repurposeable, or combinations of therapies that can force EBV-infected cells into a sensitive (e.g., dividing) state where they are druggable may be identifiable (87–89). The shock and kill anti-viral strategy.

#### 4.3.3. Immunotherapies

Immunotherapies such as anti-CD20 monoclonal antibodies are effective treatments for B cell lymphoproliferative conditions and have been re-purposed and subsequently re-designed due to efficacy in treating autoimmune conditions. However, many of these, and other immunotherapies have direct or indirect effects against EBV-infected cells, and it is unknown whether some of their beneficial effects in autoimmunity are mediated by these effects. However, in addition to this, targeted immunotherapies specifically designed to target EBV-infected cells may be possible as both lytic and latent EBV infection appears to alter the infected cell proteomics considerably including for membrane proteins which could be targets of immunotherapies (86, 90).

#### 4.3.4. Cell-Based Therapies

Cell-based therapies, finally, show great promise in treating latent EBV. This approach was successfully pioneered for the treatment of EBV post-transplant lymphoproliferative disease (a problem of poorly controlled EBV latency in the context of immunosuppression) (91–93). However, a recent clinical trial has shown exciting promise and satisfactory tolerability of *in vitro*-expanded autologous EBV-specific T cell therapies directed at a restricted subset of EBV latent proteins in persons living with secondary progressive MS (94), providing hope that this may also be a fruitful approach in treating even advanced MS.

Thus, whilst there is currently no licensed vaccine or therapy known to clear latent EBV infection, there is substantial promise on numerous fronts in this area. Understanding the pathophysiological role of EBV in MS may identify other potential routes to prevention altogether.

## 5. Conclusion

The limitations of the additive model of population genetics are well appreciated by geneticists, however, despite this there have been frequent misunderstandings and unfortunate misapplications. One result is the continuation of irreconcilable debate as to whether multiple sclerosis is primarily a genetic disease or an environmental one, even in the face of intriguing evidence that implicates EBV as a necessary cause and the discovery of much of the genetic architecture explaining the heritable variation across populations. As MS is entirely the product of gene-environment interactions it is caused (100%) both by genes and environment. Partitioning the % into the sources of variation tells us nothing about whether the causes are modifiable or not. Here it is argued that an integrated model, accepting MS susceptibility as polygenic, and that the condition may be a complication of EBV infection, offers an opportunity to understand both the role of the virus and other environmental factors and may offer new preventative strategies.

## Data Availability Statement

The original contributions presented in the study are included in the article/supplementary material, further inquiries can be directed to the corresponding author/s.

## Author Contributions

PK conceived of the presented work, wrote, and revised the manuscript.

## Funding

PK has received funding (ECAT/Wellcome fellowship and clinical lectureship) from the Wellcome Trust (223058/Z/21/Z) and funding from the Anne Rowling Regenerative Neurology Fund and the Chief Scientist Office (Scotland).

## Conflict of Interest

The author declares that the research was conducted in the absence of any commercial or financial relationships that could be construed as a potential conflict of interest.

## Publisher's Note

All claims expressed in this article are solely those of the authors and do not necessarily represent those of their affiliated organizations, or those of the publisher, the editors and the reviewers. Any product that may be evaluated in this article, or claim that may be made by its manufacturer, is not guaranteed or endorsed by the publisher.
